# Neonatal Renal Failure Following Intrauterine Exposure to an Angiotensin-Converting Enzyme Inhibitor

**DOI:** 10.7759/cureus.53833

**Published:** 2024-02-08

**Authors:** Inês Rodrigues, Carolina Quintela, Joana Jardim, Helena Pinto, Susana Pissarra, Henrique Soares, Paulo Soares

**Affiliations:** 1 Department of Pediatrics and Neonatology, Centro Hospitalar de Trás-os-Montes e Alto Douro, Vila Real, PRT; 2 Neonatal Intensive Care Unit, Department of Neonatology, Centro Hospitalar Universitário de São João, Porto, PRT; 3 Pediatric Nephrology Unit, Department of Pediatrics, Centro Hospitalar Universitário de São João, Porto, PRT; 4 Department of Gynecology-Obstetrics and Pediatrics, Faculty of Medicine, University of Porto, Porto, PRT

**Keywords:** hypotension, acute kidney injury, anuria, angiotensin-converting enzyme inhibitors, renin-angiotensin system

## Abstract

The renin-angiotensin-aldosterone system (RAAS) plays a crucial role in the normal development of the fetal kidney. Late pregnancy blockage of the RAAS, through in-utero exposure to angiotensin-converting enzyme inhibitors (ACEIs) or angiotensin II receptor blockers, is associated with poor fetal outcomes, including oligohydramnios, renal tubular dysplasia, postnatal anuric renal failure, and hypotension. The present case describes a 39-year-old primigravida that was referred to the emergency department, at 37 weeks, for the evaluation of intrauterine growth restriction and suspected coarctation of the aorta (CoA). She was medicated with enalapril since the 35th week of gestation. She delivered a male infant, weighing 2,110 g, with no apparent malformations. CoA was excluded. During his first day of life, the patient developed anuria, acute renal failure, and hypotension, requiring ionotropic support. Renal ultrasound appeared normal. Diuresis was reinitiated at 48 hours of life after continued supportive measures. Kidney function tests progressively normalized. Additional investigations revealed a low concentration of angiotensin-converting enzyme. The patient is currently 12 months old and has had a favorable evolution. This case highlights the fact that even brief exposure to enalapril in the third trimester may cause RAAS blocker fetopathy. As long-term sequelae of ACEI-exposed infants are poorly described, close follow-up of renal complications is essential. Physicians should be aware of the deleterious effects of RAAS blockers in pregnancy.

## Introduction

The integrity of the renin-angiotensin-aldosterone system (RAAS) is an essential prerequisite for the normal development of the fetal kidney [1,2]. Pharmacological blockade of the RAAS, through *in-utero* exposure to angiotensin-converting enzyme inhibitors (ACEIs) or angiotensin II receptor blockers (ARBs), compromises the normal nephrogenesis, among other deleterious effects [3]. The resulting fetopathy, usually termed *fetal RAAS blockade syndrome*, was first described in 1981 by Duminy et al. [2,4]. It is characterized by a spectrum of manifestations, ranging from transitory renal impairment to irreversible anuric renal failure and death [1,2].

Here, we report a case of ACEI fetopathy, resulting from a brief third-trimester exposure to enalapril, and a review of the literature concerning this topic.

## Case presentation

A 39-year-old woman, gravida 1 para 0, was admitted to the obstetric emergency department (ED) of a tertiary care hospital for the urgent evaluation of intrauterine growth restriction (IUGR) and suspected congenital heart disease. Her previous medical record was unremarkable. She had been receiving regular care since her first trimester. At 35 weeks of pregnancy, she was diagnosed with gestational hypertension and was since medicated with enalapril (20 mg/day). The pregnancy was otherwise uneventful.

Her first and second-trimester ultrasounds were normal. The third-trimester ultrasound (30 weeks) raised concerns for a possible cardiac malformation. Subsequent fetal echocardiograms, performed at 31 and 36 weeks, identified a probable, discrete, coarctation of the aorta (CoA). Re-evaluation at 37 weeks showed oligohydramnios and IUGR (fetal weight estimation on the second percentile by Hadlock), with pathological umbilical blood flow on Doppler study. There was no evidence of preterm rupture of membranes. She was referred to the ED for urgent evaluation. On admission, cardiotocography showed signs of fetal distress, requiring an emergency cesarean section. The woman gave birth to a male newborn, weighing 2,110 g (third percentile, according to the Intergrowth-21st charts), measuring 45.6 cm (11th percentile), and with a head circumference of 34.5 cm (88th percentile). His Apgar scores were 8 and 9, at one and five minutes, respectively.

The neonate was admitted to the neonatal intensive care unit for close monitoring. He had a normal physical examination and was hemodynamically stable, with a mean blood pressure (BP) of 39 mmHg, and no BP differential between the upper and lower extremities. An echocardiogram was performed and excluded any cardiac anomalies. Through his first hours of life, the newborn was clinically stable and had a registered micturition of 20 mL. There was no urine output past 15 hours of life, despite increasing parenteral nutrition to a volume load of 120 mL/kg/day. At 24 hours of life, the patient remained anuric and developed severe hypotension (mean BP: 20 mmHg), with poor response to fluids. Dopamine was initiated and gradually titrated to 15 µg/kg/minute. Initial laboratory findings revealed metabolic acidosis (pH: 7.28, HCO_3_: 17.8 mmol/L, lactate: 4.2 mmol/L), hyponatremia (128 mmol/L), a raised serum creatinine of 200.7 μmol/L (2.27 mg/dL), and serum urea of 10.8 mmol/L (65 mg/dL). His sepsis workup was negative, and he was not receiving antibiotics or any other nephrotoxic agents.

Renal ultrasound with Doppler scanning showed normal-sized kidneys, with adequate blood flow, and an empty bladder. After a dose of furosemide (1 mg/kg), an albumin bolus (0.5 g/kg), and continued dopamine infusion, diuresis was reinitiated at 48 hours of life. In the following days, the patient maintained an adequate urine output (>1 mL/kg/hour). BP normalized, and dopamine was gradually reduced and stopped on the sixth day of life.

Urea peaked at 22 mmol/L (132 mg/dL) on day three, and serum creatinine at 371.3 μmol/L (4.20 mg/dL) on day five, with subsequent normalization. Additional investigations (day six) revealed a low concentration of angiotensin-converting enzyme (ACE <8 U/L, normal value: 20-70 U/L). Urine analysis showed glucosuria, albuminuria (39.1 mg/dL), high urinary β2-microglobulin (11,900 µg/L, normal value: <300 µg/L), as well as an increased spot urine protein-to-creatinine ratio (1.7 mg/mg). The patient was discharged on the 12th day of life.

**Figure 1:**
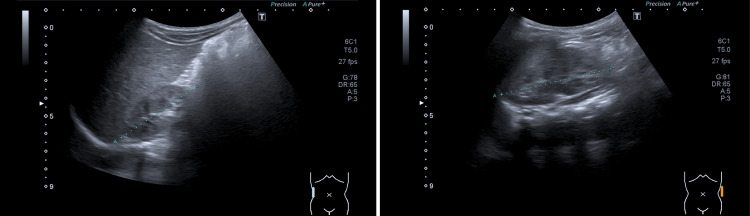
Bilateral renal ultrasound performed at 12 months of age. Normal-sized kidneys with preserved corticomedullary differentiation.

Currently, at 12 months of age, the patient has adequate neurodevelopment and growth. Renal ultrasound at three months revealed normal-sized kidneys, with diffuse cortical hyperechogenicity and reduced corticomedullary differentiation. These alterations were not seen in subsequent ultrasounds (Figure 1). Serum creatinine and urea remain within the normal range, and initial alterations seen on urinalysis resolved. He maintains regular follow-up.

## Discussion

Hypertension is highly prevalent and affects 7.7% of reproductive-aged women [5]. Hypertensive disorders of pregnancy, which include preexisting and gestational hypertension, complicate up to 10% of all pregnancies, representing an important cause of maternal-fetal morbidity [5]. The National Institute for Health and Care Excellence currently recommends ACEIs and ARBs as first-line treatments for hypertension in adults, including women of *childbearing potential*, stating, however, that both medications are contraindicated during pregnancy [6]. Given the rising prevalence of chronic hypertension in women of reproductive age, as well as the increasing birth rate among advanced maternal-age women (which is associated with a higher risk of both essential and gestational hypertension), the inadvertent use of RAAS blockers in pregnancy is likely to increase.

Fetal circulation is characterized by low pressures, requiring high angiotensin II levels to maintain adequate renal perfusion [2]. Inhibition of the ACE results in hypoperfusion and ischemia, compromising the normal tubular development [1,2]. This leads to oligohydramnios, which is often the first sign of RAAS blockade fetopathy. After birth, anuria and hypotension develop [7].

A review of 118 cases of exposure to ACEI concluded that the most common complications were renal failure (23%), anuria (20%), oligohydramnios (19%), death (18%), hypotension (17%), and IUGR (15%) [2]. Fetal anuria is associated with pulmonary hypoplasia and respiratory distress, which are also frequent manifestations of the RAAS blockade syndrome [1,2,7]. Another common finding is hypocalvaria, which is attributed to hypotension and reduced cranial vascularization [1,3,7].

In this case, although there was only a two-week exposure to enalapril, the newborn exhibited some of the most frequent manifestations of the RAAS blocker fetopathy, including oligohydramnios, postnatal hypotension, and acute anuric renal failure, with a favorable course. To our knowledge, this is the first report of RAAS blocker fetopathy described after such a brief exposure to an ACEI. In fact, the time of exposure to RAAS blockers seems to determine the severity of symptoms. A review of 190 newborns exposed to either ACEIs (n = 89) or ARBs (n = 101) concluded that RAAS fetopathy was not observed if the exposure ceased before 20 weeks [7]. Many other reports also consistently document poorer outcomes in newborns exposed in the second and third trimesters versus those exclusively exposed in the first [1,2].

Information about the long-term follow-up of these patients is scarce. In the anteriorly mentioned review, follow-up was only available in 26 children (14 of whom had been exposed to an ACEI) [2]. Renal complications were the most frequent problem (which included renal failure in 23% of patients, and hypertension in 15%), followed by neurodevelopmental delay and failure to thrive [2]. Up until now, the presented patient has not experienced any of these complications.

## Conclusions

Brief third-trimester enalapril exposure is associated with RAAS blocker fetopathy and subsequent acute anuric renal failure. Long-term sequelae of ACEI-exposed infants are poorly described, and, therefore, close follow-up of renal complications is essential. Physicians from different fields should be aware of the deleterious effects of RAAS blockers in pregnancy.
